# Tobacco Use and Cardiovascular Disease among American Indians: The Strong Heart Study

**DOI:** 10.3390/ijerph7103816

**Published:** 2010-10-25

**Authors:** June E. Eichner, Wenyu Wang, Ying Zhang, Elisa T. Lee, Thomas K. Welty

**Affiliations:** 1 Department of Biostatistics and Epidemiology, University of Oklahoma Health Sciences Center, CHB Rm. 354, 801 NE 13th St., Oklahoma City, OK 73104, USA; 2 Center for American Indian Health Research, University of Oklahoma Health Sciences Center, 801 NE 13th St., Oklahoma City, OK 73104, USA; E-Mails: wenyu-wang@ouhsc.edu (W.W.); ying-zhang4@ouhsc.edu (Y.Z.); elisa-lee@ouhsc.edu (E.T.L.); 3 Missouri Breaks Industries Research, Inc., HCR 64 Box 52, Timber Lake, SD 57656, USA; E-Mail: twelty@earthlink.net (T.K.W.)

**Keywords:** American Indians, tobacco use, cardiovascular disease, Strong Heart Study

## Abstract

Tobacco use among American Indians has a long and complicated history ranging from its utilization in spiritual ceremonies to its importance as an economic factor for survival. Despite this cultural tradition and long history, there are few studies of the health effects of tobacco in this population. The Strong Heart Study is a prospective observational study of cardiovascular disease (CVD) in 13 American Indian tribes in Arizona, Oklahoma, and North and South Dakota with 4,549 participants. Baseline examinations were followed by two examinations at regular intervals and 16 years of morbidity and mortality follow-up. Hazard ratios (HRs) for non-fatal CVD for current smokers *vs.* non-smokers after adjusting for other risk factors were significant in women (HR = 1.94, 95% CI 1.54 to 2.45) and men (HR = 1.59, 95% CI 1.16 to 2.18). Hazard ratios for fatal CVD for current smokers *vs.* non-smokers after adjusting for other risk factors were significant in women (HR = 1.64, 95% CI 1.04 to 2.58), but not in men. Individuals who smoked and who were diagnosed with diabetes mellitus, hypertension or renal insufficiency were more likely to quit smoking than those without these conditions. On average, American Indians smoke fewer cigarettes per day than other racial/ethnic groups; nevertheless, the ill effects of habitual tobacco use are evident in this population.

## 1. Introduction

Tobacco has a long and complicated history for American Indians, having been used for religious and therapeutic purposes early on and then later cultivated and traded with European settlers [1]. It is still used today in cultural practices, such as burial offerings, for spiritual protection, or as a gift [1]. Partly due to this history, a mystique surrounding tobacco and American Indians has evolved over time. American Indians have high rates of habitual tobacco use, particularly smoking, although chewing and dipping are also practiced. Smoking rates among adult American Indians are the highest for any race/ethnicity in the United States. In 1999–2001, 40.4% of American Indians aged 18 years and above reported cigarette use during the preceding month compared to the national average of 26.5% [2–4]. In recent times the establishment of smoke shops which function as a source of livelihood in some Indian communities has also promoted this habit. Relatively little has been reported on the cardiovascular health effects of tobacco use among American Indians.

Cardiovascular disease (CVD) risk factors have been studied extensively in longitudinal studies, including the Framingham Heart Study, studies from the Pooling Project, Multiple Risk Factor Intervention Trial (MRFIT), Atherosclerosis Risk in Communities (ARIC), and internationally in the World Health Organization (WHO) sponsored MONICA Study (Monitoring Trends and Determinants in Cardiovascular Diseases) [5–12]. These studies and others with prospective designs have confirmed that an elevated cholesterol level, high blood pressure and cigarette smoking are major risk factors for coronary heart disease (CHD), and high blood pressure and smoking are risk factors for all categories of stroke [13,14]. These studies provide interesting comparisons to the Strong Heart Study, a prospective study of CVD in American Indians. American Indians share the same risk factors as other populations, but have an increased susceptibility to and high prevalence of another important CVD risk factor—type 2 diabetes. In addition to the high rates of diabetes found among American Indians, there are also very high rates of tobacco use among some tribes [15–17]. When looking at multiple CVD risk factors simultaneously, Strong Heart Study investigations found smoking to be a risk factor for some subcategories of CVD but not others [18–21]. Tobacco use was not a focus of any of these studies. The present study focuses on tobacco use and its role as a contributor to CVD among participants in the Strong Heart Study.

## 2. Experimental Section

### 2.1. Study Population

The Strong Heart Study is a prospective study of cardiovascular risk factors, and CVD morbidity and mortality among 13 American Indian tribes/communities residing in Arizona, Oklahoma and North and South (N/S) Dakota. The Strong Heart Study protocol was reviewed and approved by the institutional review boards (IRBs) of the participating institutions, the 13 participating tribes, and the Indian Health Service IRBs for the three geographic areas wherein the tribes reside. Each participant gave informed consent. The initial cohort of 4,549 (2,703 females and 1,846 males) participants were aged 45 to 74 at first examination. Roughly 1,500 individuals were included from each center, among whom 4,293 were free from CVD and had known tobacco exposure status at the baseline examination. Baseline assessment was conducted between July 1989 and January 1992 with follow-up examinations at regular intervals. Each participant has been tracked for health outcomes, especially non-fatal and fatal CVD. The study design, method and participation rates have been explained in earlier Strong Heart Study publications [22,23].

### 2.2. Risk Factor Assessments and Definitions

The baseline examination consisted of a personal interview and a physical examination. Interview questions during this examination included information on demographics, family history, health habits, medical history and medications. After a five-minute rest, blood pressure was taken three times, and the mean of the last two measurements was used to estimate blood pressure. Anthropometric measurements of height and weight were taken, and body mass index (BMI) calculated as weight (kg) divided by height squared (m^2^). A morning urine specimen was obtained for measurements of albumin and creatinine. After a 12-hour overnight fast, blood samples were obtained for laboratory measurements. A 75 gram oral glucose tolerance test was also performed [24].

Use of smoked tobacco was categorized. A current smoker was defined as a person who smoked more than 100 cigarettes in his/her lifetime and was still smoking. An ever smoker was defined as a person who smoked more than 100 cigarettes in his/her lifetime but was no longer smoking. A non-smoker was defined as a person who had not smoked more than 100 cigarettes in his/her lifetime and was not currently smoking. Diabetes mellitus was classified according to the American Diabetes Association (ADA) criteria, with diabetes defined as taking insulin or oral anti-diabetic medication or having a fasting glucose concentration ≥126 mg/dL. A person was considered to have hypertension if he/she had a systolic blood pressure ≥140 mmHg or a diastolic blood pressure ≥90 mmHg, or if he/she was taking antihypertensive medication. Renal problems were assessed by the ratio of albumin to creatinine in the urine. Microalbuminuria and macroalbuminuria were defined as a ratio of albumin to creatinine of 30–299 mg/g and ≥300 mg/g, respectively.

### 2.3. Outcome Variables

After the baseline assessment, reexaminations were conducted in 1993–1995 and 1998–1999. During each examination, a 12-lead ECG and medical history, including the Rose questionnaire for angina pectoris, were obtained [25]. Deaths of study participants were identified through obituaries in local newspapers, tribal and Indian Health Service hospital records and by direct contact of study personnel with the participant’s family or other informant. Cause of death was assessed through death certificates, autopsy reports, medical records and informant interviews, as reported previously [26]. Nonfatal CVD included definite myocardial infarction (MI), definite CHD and definite stroke. Fatal CVD included definite fatal MI, definite sudden death due to CHD, definite fatal CHD and possible fatal CHD, definite fatal stroke and possible fatal stroke. Events occurring till December 31, 2005 were included in the analysis. Mortality follow-up was 99.8% complete.

### 2.4. Statistical Methods

Descriptive statistics (means, standard deviations and proportions) stratified by smoking status were calculated for men and women separately. Cox proportional hazards models with competing risks were employed to estimate the independent contribution of current smoking and ever smoking in men and women separately with time to first non-fatal and fatal CVD outcome [27]. Each proportional hazards model included relevant risk factors to control for possible confounding. SAS 9.1 (Cary, NC) was used for analysis.

## 3. Results

**Figure 1.**
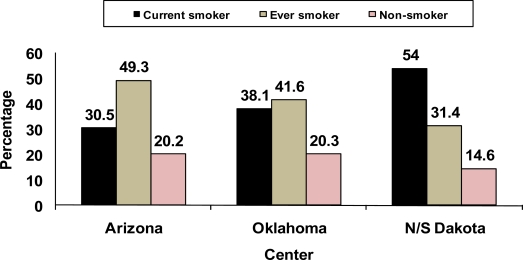
Cigarette smoking among American Indian men by center, Strong Heart Study, 1989–1992. Current smoker was defined as a person who smoked more than 100 cigarettes during his/her lifetime and answered ‘yes’ to the question “Do you smoke now?” An ever smoker was defined as a person who smoked more than 100 cigarettes in his/her lifetime but was not smoking now. A non-smoker was defined as a person who had not smoked more than 100 cigarettes in his/her lifetime and was not currently smoking.

**Figure 2.**
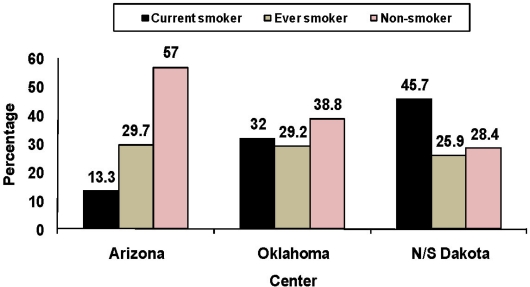
Cigarette smoking among American Indian women by center, Strong Heart Study, 1989–1992. Current smoker was defined as a person who smoked more than 100 cigarettes during his/her lifetime and answered ‘yes’ to the question “Do you smoke now?” An ever smoker was defined as a person who smoked more than 100 cigarettes in his/her lifetime but was not smoking now. A non-smoker was defined as a person who had not smoked more than 100 cigarettes in his/her lifetime and was not currently smoking.

Variation is seen in the smoking rates among American Indians at the Strong Heart Study sites as seen in Figures 1 and 2. The prevalence of current smoking among the Oklahoma American Indian population was higher than that of Arizona American Indians, but less than that of the N/S Dakota American Indians. These rates were statistically different among the study sites for both males and females (*P* ≤ 0.01). This exemplifies the variation seen regionally in the U.S. among American Indians.

Prevalence of smoking was significantly higher among males than females with roughly 40% reporting current smoking. The Arizona site had the lowest smoking prevalence with only about one in five reporting current smoking, and 43.8% reporting no smoking. The Dakota site had the highest prevalence of smoking with roughly one-half of the participants reporting current and 22.7% reporting no smoking. The Oklahoma site had an intermediate prevalence proportion with roughly one-third reporting current and one-third no smoking. Also, smoking cessation rates were highest in Arizona and lowest in the Dakotas.

**Figure 3.**
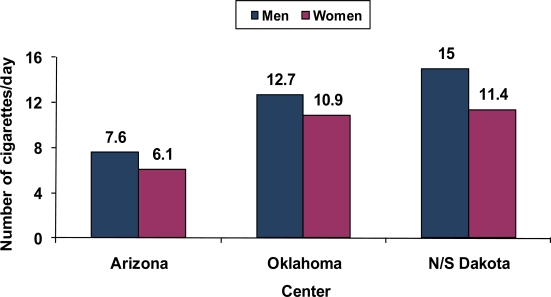
Number of cigarettes smoked per day among current smokers by center, Strong Heart Study, 1989–1992. Current smoker was defined as a person who smoked more than 100 cigarettes during his/her lifetime and answered ‘yes’ to the question “Do you smoke now?”.

**Figure 4.**
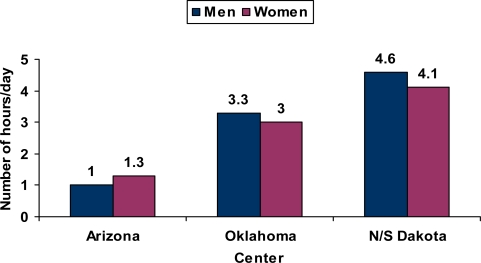
Mean number of hours per day of exposure to environmental tobacco smoke.

The mean number of cigarettes smoked per day among current users was less in Arizona and higher in N/S Dakota as compared to Oklahoma. Figure 3 provides the mean numbers of cigarettes smoked per day at each site [22]. Even in N/S Dakota, the average for men was less than 20 cigarettes per day. At each center, women on average smoked fewer cigarettes per day than men. As might be expected, exposure to environmental tobacco smoke followed a similar pattern with greater exposure in N/S Dakota, less exposure in Oklahoma and the least exposure to environmental tobacco smoke in Arizona (Figure 4) [22].

Baseline characteristics of participants stratified by smoking status are shown in Table 1. About one-third of the cohort was current smokers, one-third was ever-smokers and one-third was non-smokers. Covariates were not evenly distributed among the three smoking classifications (Table 1). Mean age was lowest among current smokers both male (54.7 years) and female (54.9 years). Body mass index (BMI [wt(kg)/ht(m)^2^]) was lowest for current smokers of both genders, *i.e.*, mean male BMI = 28.6 and mean female BMI = 30. The means of ever and never smokers were similar, 30.8 and 30.6 for ever and never male smokers respectively and 32.7 and 31.9 for ever and never female smokers respectively. The mean low density lipoprotein (LDL) cholesterol level was higher for current smokers compared to non-smokers for women and men though the differences were not statistically significant for men.

For women only 36% of current smokers had diabetes compared to over 50% of ever and never smokers. The same pattern was seen in men with 33% of current smokers having diabetes, and 47% of ever smokers and 44% of never smokers with diabetes. Hypertension was less common in both males and females who were current smokers. Current smoking status is statistically significantly associated with less diabetes mellitus, less hypertension, less albuminuria, younger age, lower BMI, lower HDL cholesterol and higher LDL cholesterol.

Tables 2 and 3 provide hazard ratios for non-fatal and fatal CVD for ever smokers and current smokers compared to non-smokers after adjusting for other known risk factors. Compared with non-smokers, being a current smoker significantly increased the risk of non-fatal CVD by 94% in women after adjusting for age, BMI, LDL cholesterol, HDL cholesterol, quartile of physical activity, presence of hypertension, diabetes and macro and microalbuminuria (Table 2). Compared with non-smokers, being a current smoker significantly increased the risk of fatal CVD by 64% for women (Table 3). For men, compared to non-smokers, neither being an ever nor current smoker reached statistical significance in the adjusted hazard ratios for fatal CVD. However, compared to male non-smokers, being a current smoker significantly increased the risk for non-fatal CVD by 59%.

Table 4 displays the disease status stratified percentages of participants without CVD who quit smoking before the Phase 2 exam and remained free of CVD at the end of 2005. Participants with diabetes mellitus, hypertension or macro/microalbuminuria detected at the Phase 1 exam were significantly more likely to quit smoking than those without these conditions.

## 4. Discussion and Conclusions

There has been a downward trend in tobacco use among American Indians with the smoking proportion dropping by 10% between 1987–1990 and 1994–1995 [2]. Use has continued to decline in the first decade of 2000 [28–30]. These trends should beneficially contribute to the health status of American Indians. Despite these trends, previous studies of selected American Indian tribes have found that smoking is not perceived as a risk factor for CVD [31,32].

Tobacco use is a major contributor to disease and death [33–35]. Among the 361,662 men screened for the MRFIT, cigarette smoking was an important risk factor for all-cause, CHD, stroke and cancer mortality [36]. Based on the Framingham Heart Study, Mamun *et al.* reported that male and female non-smokers live 8.66 and 7.59 years longer than smokers and have more years free of CVD [37]. There is little argument that tobacco use is a habit with considerable health risks attached.

Among American Indians higher smoking rates were seen in younger individuals. In addition, smoking rates decreased with advancing age, and the mean age of current smokers was less than that of non-smokers [4]. As individuals aged and developed other CVD risk factors, they were more likely to quit smoking. This has been reported by Zhang *et al.* [38] as well. Even though current smoking was associated with a reduced presence of other CVD risk factors, both male and female smokers had higher LDL cholesterol levels. An autopsy study has shown that smoking is associated with fatty streaks and raised lesions in the intimal surfaces of arteries in youth, providing further evidence for the contribution of smoking to atherogenesis [39].

In a number of studies, current smoking increased the risk for CHD by 50% to 80%, and the risk appeared to be more evident in women than in men [40]. In the Strong Heart Study, American Indian women showed the adverse effects of smoking on CVD risk more noticeably than men. Despite the high prevalence of tobacco use, American Indians smoke fewer cigarettes each day compared to whites, and this may partly attenuate its impact on risk [2,4,28]. Based on the MRFIT, there was a graded risk of vascular disease with greater use associated with a greater CVD death rate in white men [41]. On the other hand, the Copenhagen City Heart Study of 12,149 men and women showed that smoking as little as 3–5 grams of tobacco per day (equivalent to 3–5 cigarettes per day) or not inhaling the smoke increased the risk of MI and all-cause mortality with a higher relative risk (RR) in women (RR = 2.14) than in men (RR = 1.86). In men, increased risks were seen when using 6–9 grams per day (equivalent to 6–9 cigarettes per day) with RRs of MI and all-cause mortality of 2.10 and 1.76 respectively [42].

In the Strong Heart Study, smoking behavior differed based on risk factor stratification. Compared to ever smokers, both male and female current smokers had a lower prevalence of diabetes, hypertension, and albumin/creatinine ratio ≥300 mg/g(%). Greater proportions of those who smoked and had diabetes, hypertension or renal insufficiency quit smoking than those who smoked and did not have co-morbid conditions. The risk factors of diabetes, hypertension and albuminuria were statistically significant for both men and women and for both non-fatal and fatal CVD (HRs ranging from 1.33 to 4.60). It has been demonstrated that one or more major risk factors—an elevated cholesterol level, hypertension, cigarette smoking, or diabetes—are antecedent risks in over 85% of CHD events, and efforts to minimize these risk factors should be continued in all populations [43].

The cultural and economic role of tobacco in American Indian communities complicates but does not prohibit prevention efforts. For thousands of years tobacco has played an integral role in some American Indian traditions. The problem is with abuse of tobacco and not with ceremonial or traditional use. There are participants in both the Strong Heart Study and Cherokee Diabetes Study who reported being non-smokers but who did use tobacco for ceremonial or sacramental purposes [4]. Loss of revenue from smoke shops in impoverished communities may bring an economic hardship to some; nevertheless, progress is being made in health literacy. Many tribal council meetings, once conducted in smoke filled rooms, are now smoke free [1]. Prevention and cessation programs are available though Indian Health Service programs and tribal health services, though more are needed in these communities.

## Figures and Tables

**Table 1.** Baseline characteristics by gender and smoking status, Strong Heart Study, 1989–1992.

Variable	**Males (n = 1689)**				**Females (n = 2604)**			
Mean (S.D.) or %	Never (n = 308) (18%)	Ever (n = 682) (40%)	Current (n = 699) (41%)	P-value	Never (n = 1093) (42%)	Ever (n = 738) (28.6%)	Current (n = 773) (30%)	P-value
Age (years)	55.7 (7.8)	55.9 (7.9)	54.7 (7.4)	0.016	57.6 (7.9)	56.5 (7.9)	54.9 (7.5)	<0.001
BMI*	30.6 (6.4)	30.8 (6.1)	28.6 (5.3)	<0.001	31.9 (6.7)	32.7 (6.3)	30.0 (6.3)	<0.001
LDL cholesterol* (mg/dl)	117.0 (32.0)	118.7 (34.3)	120.6 (31.4)	0.240	115.5 (32.1)	116.8 (32.0)	121.9 (33.9)	<0.001
HDL cholesterol* (mg/dl)	44.0 (13.8)	42.3 (12.2)	43.8 (14.9)	0.060	48.4 (13.1)	47.9 (12.9)	46.8 (13.1)	0.047
Diabetes Mellitus* (%)	44	47	33	<0.001	52	53	36	<0.001
Hypertension* (%)	43	46	30	<0.001	42	41	28	<0.001
Albumin/creatinine ratio 30–299 mg/g (%)	18	18	17	0.762	21	22	15	<0.001
Albumin/creatinine ratio ≥ 300 mg/g (%)	12	11	6	0.002	12	14	8	<0.001
Physical Activity (minutes in past week)	87.7 (96.7)	91.6 (101.1)	99.6 (100.5)	0.150	49.6 (63.0)	60.4 (71.9)	69.6 (73.7)	<0.001

* BMI, body mass index [weight (kg)/height (m)^2^]; LDL cholesterol, low density lipoprotein cholesterol; HDL cholesterol, high density lipoprotein cholesterol; diabetes mellitus defined as taking insulin or oral anti-diabetic medication or having a fasting glucose concentration ≥126 mg/dL; hypertension defined as having a systolic blood pressure ≥140 mmHg or a diastolic blood pressure ≥90 mmHg or taking antihypertensive medication.

**Table 2.** Hazard ratios of risk factors related to non-fatal cardiovascular disease in American Indian men and women, Strong Heart Study, 1989–2005.

**Risk Factor**	**Males (n = 1,689)**			**Females (n = 2,604)**		
	Hazard Ratio*	**95% CI**		**Hazard Ratio**	**95% CI**	
Current smoker†	**1.59**	1.16	2.18	**1.94**	1.54	2.45
Ever smoker†	1.11	0.81	1.52	1.23	0.97	1.57
Age	**1.05**	1.03	1.06	**1.03**	1.02	1.04
BMI†	1.00	0.98	1.02	1.00	0.98	1.02
LDL cholesterol†	**1.01**	1.01	1.01	**1.01**	1.00	1.01
HDL cholesterol†	0.99	0.98	1.00	0.99	0.98	0.99
2nd Quartile physical activity†	1.10	0.81	1.48	0.88	0.67	1.15
3rd Quartile of physical activity†	0.91	0.67	1.24	1.01	0.77	1.31
4th Quartile of physical activity†	1.08	0.81	1.45	0.87	0.66	1.15
Diabetes mellitus†	**1.61**	1.27	2.05	**2.35**	1.87	2.97
Hypertension†	**1.36**	1.10	1.69	**1.33**	1.08	1.64
Macroalbuminuria†	**1.82**	1.28	2.58	**2.25**	1.70	2.97
Microalbuminuria†	1.28	0.97	1.69	**1.45**	1.14	1.85

* Cox proportional hazards models with competing risks were used to estimate the independent contribution of current smoking and ever smoking to non-fatal cardiovascular outcome. Bolded ratios were statistically significant (p ≤ 0.05).

† Current smoker was defined as smoking at least 100 cigarettes during his/her lifetime and answering “yes” to the question, “Do you smoke now?; ever smoker was defined as smoking more than 100 cigarettes in his/her lifetime but not currently smoking; BMI, body mass index [weight (kg)/height (m)^2^]; LDL cholesterol, low density lipoprotein cholesterol; HDL cholesterol, high density lipoprotein cholesterol; physical activity (minutes in past week) was calculated from all leisure-time exercise and activities by a questionnaire designed and validated for this study; diabetes mellitus was defined as taking insulin or oral anti-diabetic medication or having a fasting glucose concentration ≥126 mg/dl; hypertension was defined as having a systolic blood pressure ≥140 mmHg or a diastolic blood pressure ≥90 mmHg or taking antihypertensive medication; macroalbuminuria was defined as a ratio of albumin to creatinine of ≥300 mg/g; and microalbuminuria was defined as a ratio of albumin to creatinine of 30–299 mg/g.

**Table 3.** Hazard ratios of risk factors related to fatal cardiovascular disease in American Indian men and women, Strong Heart Study, 1989–2005.

**Risk Factor**	**Males (n = 1,689)**			**Females (n = 2,604)**		
	Hazard Ratio*	**95% CI**		**Hazard Ratio**	**95% CI**	
Current smoker†	1.02	0.64	1.64	**1.64**	1.04	2.58
Ever smoker†	0.61	0.38	0.99	1.33	0.88	2.02
Age	**1.08**	1.05	1.10	**1.07**	1.04	1.09
BMI†	1.00	0.96	1.03	0.96	0.93	0.99
LDL cholesterol†	**1.00**	1.00	1.01	**1.00**	1.00	1.01
HDL cholesterol†	1.01	0.99	1.02	0.99	0.97	1.00
2nd Quartile physical activity†	1.10	0.68	1.80	0.67	0.43	1.04
3rd Quartile of physical activity†	0.91	0.55	1.50	0.49	0.30	0.79
4th Quartile of physical activity†	0.65	0.38	1.13	0.39	0.23	0.68
Diabetes mellitus†	**2.12**	1.39	3.24	**2.71**	1.67	4.37
Hypertension†	**1.65**	1.13	2.41	**1.79**	1.20	2.67
Macroalbuminuria†	**2.74**	1.60	4.67	**4.60**	2.88	7.34
Microalbuminuria†	1.52	0.96	2.41	**1.76**	1.09	2.84

* Cox proportional hazards models with competing risks were used to estimate the independent contribution of current smoking and ever smoking to fatal cardiovascular outcome. Bolded ratios were statistically significant (p ≤ 0.05).

† Current smoker was defined as smoking at least 100 cigarettes during his/her lifetime and answering “yes” to the question, “Do you smoke now?; ever smoker was defined as smoking more than 100 cigarettes in his/her lifetime but not currently smoking; BMI, body mass index [weight (kg)/height (m)^2^]; LDL cholesterol, low density lipoprotein cholesterol; HDL cholesterol, high density lipoprotein cholesterol; physical activity (minutes in past week) was calculated from all leisure-time exercise and activities by a questionnaire designed and validated for this study; diabetes mellitus was defined as taking insulin or oral anti-diabetic medication or having a fasting glucose concentration ≥126 mg/dl; hypertension was defined as having a systolic blood pressure ≥140 mmHg or a diastolic blood pressure ≥90 mmHg or taking antihypertensive medication; macroalbuminuria was defined as a ratio of albumin to creatinine of ≥300 mg/g; and microalbuminuria was defined as a ratio of albumin to creatinine of 30–299 mg/g.

**Table 4.** Smoking cessation rates for current smokers according to disease status and smoking status at Phase 1 exam who did not have CVD at the end of 2005, Strong Heart Study, 1989–2005.

Disease*	Smoking Cessation Rate (%)**	**p-value**†
Diabetes Mellitus*		
Yes	23.48	0.0012
No	15.40	
Hypertension*		
Yes	22.78	0.0139
No	16.43	
Renal Insufficiency*		
Yes	26.34	0.0006
No	16.22	

* Current smoker was defined as smoking at least 100 cigarettes during his/her lifetime and answering “yes” to the question, “Do you smoke now?; ever smoker was defined as smoking more than 100 cigarettes in his/her lifetime but not currently smoking; BMI, body mass index [weight (kg)/height (m)^2^]; LDL cholesterol, low density lipoprotein cholesterol; HDL cholesterol, high density lipoprotein cholesterol; physical activity (minutes in past week) was calculated from all leisure-time exercise and activities by a questionnaire designed and validated for this study; diabetes mellitus was defined as taking insulin or oral anti-diabetic medication or having a fasting glucose concentration ≥ 126 mg/dl; hypertension was defined as having a systolic blood pressure ≥140 mmHg or a diastolic blood pressure ≥90 mmHg or taking antihypertensive medication; macroalbuminuria was defined as a ratio of albumin to creatinine of ≥300 mg/g; and microalbuminuria was defined as a ratio of albumin to creatinine of 30–299 mg/g. Diseases were detected at Phase 1 exam.

** Percentage of participants who quit smoking before Phase 2 exam.

† p-value from testing rate differences between diseased and non-diseased.
